# Ocular Surface Infection by SARS-CoV-2 in COVID-19 Pneumonia Patients Admitted to Sub-Intensive Unit: Preliminary Results

**DOI:** 10.3390/microorganisms10020347

**Published:** 2022-02-02

**Authors:** Mario Troisi, Carla Zannella, Salvatore Troisi, Maddalena De Bernardo, Massimiliano Galdiero, Gianluigi Franci, Nicola Rosa

**Affiliations:** 1 Department of Medicine, Surgery and Dentistry Scuola Medica Salernitana, University of Salerno, 84081 Baronissi, Italy; mdebernardo@unisa.it (M.D.B.); gfranci@unisa.it (G.F.); nrosa@unisa.it (N.R.); 2 Department of Experimental Medicine, University of Campania Luigi Vanvitelli, 80138 Naples, Italy; carla.zannella@unicampania.it (C.Z.); massimiliano.galdiero@unicampania.it (M.G.); 3 Ophtalmologic Unit AOU San Giovanni di Dio e Ruggi D’Aragona Scuola Medica Salernitana, 84125 Salerno, Italy; salvatore.troisi@gmail.com

**Keywords:** conjunctival swab, COVID-19 diagnosis, eye infection, ocular transmission SARS-CoV-2, RT-PCR, viral keratoconjunctivitis

## Abstract

The aim of the present study is to check the relationship between virus detection on the conjunctival swabs by RT-PCR and the systemic and ocular clinical data, treatments, and to the modalities of administration of supplemental oxygen. The SARS-CoV-2 RNA reverse-transcriptase PCR assay of conjunctival brushing samples and the corneal/conjunctival clinical findings were evaluated in 18 eyes of 9 consecutive patients admitted to the COVID-19 Sub-intensive Unit of Salerno Hospital University, Italy. Conjunctival swabs were positive for SARS-CoV-2 in 13 eyes of 7 patients; corneal epithelial defects were detected in 9 eyes. The seven patients with ocular involvement from SARS-CoV-2 had undergone treatment with a full-face mask or oxygen helmet in the last week, while the two subjects with negative conjunctival swabs had been treated with high flow nasal cannula. The positivity to the conjunctival test for SARS-CoV-2 was higher (72%) than that reported in the literature (10–15%) and related in all cases to the use of facial respiratory devices. These results suggest that exposure of unprotected eyes to aerosols containing high concentrations of SARS-CoV-2 could cause a keratoconjunctival viral infection. Further studies are needed to verify the causal link with the use of respiratory facial devices in patients suffering from COVID-19 pneumonia.

## 1. Introduction

Severe acute respiratory syndrome coronavirus 2 (SARS-CoV-2) is a highly transmissible single-stranded RNA-virus that spreads mainly through person-to-person contact via respiratory droplets or through contact with contaminated surfaces or objects from an asymptomatic, presymptomatic, and symptomatic infected person [1]. SARS-CoV-2 causes a worldwide sudden and substantial increase in hospitalizations for pneumonia with a multiorgan disease, named coronavirus disease 2019 (COVID-19) [1]. To limit the spread of SARS-CoV-2, many governments enforced “lockdowns” of varying degrees, and several medical societies worldwide issued recommendations regarding the cessation of routine clinical and surgical duties. Among these, ophthalmologic units have been significantly affected by these changes because they largely deal with elective surgeries [2]. Many studies show that COVID-19 is potentially complicated by ocular involvement, especially with kerato-conjunctival inflammation [3,4]. The pathogenetic mechanisms of ocular infection in humans have not been widely studied. However it was reported that the eye may constitute not only a potential site of virus replication but also an alternative transmission route of the virus [4]. This could be caused by the increased expression of the angiotensin 2 converting enzyme (ACE 2) receptors that are used by the virus to enter the epithelial cells of the respiratory surface and conjunctiva [5]. In fact, the expression of ACE 2 receptors has been demonstrated in the epithelium of human conjunctiva but not in the stroma, using single-cell RNA–sequencing; moreover, in some cases, red-eye or conjunctivitis may be the first sign of the infection [6]. There is limited evidence regarding SARS-CoV-2 transmission through other routes, such as the fecal–oral route, which relies upon the detection of the virus in different types of tissue and fluids, including sputum, blood, and feces [3]. Diagnosis is made by detection of the viral RNA via reverse-transcription polymerase chain reaction (RT-PCR) testing; the false-negative test results may occur in up to 20 to 67% of patients, depending on the quality and timing of testing [7]. The aim of this study was to evaluate the infection of the ocular surface by SARS-CoV-2 in patients admitted to the Sub-intensive Unit of Salerno Hospital University (Salerno, Italy) for COVID-19 pneumonia under supplemental oxygen treatment. The results of the virus detection on the conjunctival swabs by RT-PCR are related to the systemic and ocular clinical data, treatments, and to the modalities of administration of supplemental oxygen.

## 2. Materials and Methods

### 2.1. Samples Collection

Nine consecutive patients suffering from COVID-19 pneumonia underwent brushing of the lower and upper tarsal conjunctiva of both eyes. Thirty minutes before the collection of the conjunctival swab, conjunctival evaluation was carried out using Efron grading scale system [8]; subsequently, staining with sodium fluoresceinate 2% and corneal examination was performed using cobalt blue filter of binocular indirect ophthalmoscope illumination system (Keeler Ltd., Windsor, UK). 

All the patients were hospitalized, requiring intensive care and supplemental oxygen. Inclusion criteria: nasopharyngeal swab positive in the last 48 h, no ocular therapies in progress, and COVID-19 symptoms with acute respiratory failure for at least seven days.

### 2.2. RT-PCR

The samples taken were examined by RT-PCR assay. The samples taken were examined by RT-PCR assay. RNA extraction was performed by TRIzol (Thermo Fisher Scientific, Waltham, MA, USA); RNA samples were quantified at Nanodrop (NanoDrop 2000, Thermo Fisher Scientific) and retrotranscribed to cDNA by the 5× All-In-One RT MasterMix kit (Applied Biological Materials, Richmond, B.C., Canada). Then, 2 µL of cDNA were amplified for each sample, the level of spike protein (forward, AGGTTGATCACAGGCAGACT; reverse, GCTGACTGAGGGAAGGAC) was analyzed and expressed using the 2-ΔΔCt method. Relative target threshold cycle (Ct) values were normalized to Glyceraldehyde 3-phosphate dehydrogenase (GAPDH) (forward, CCTTTCATTGAGCTCCAT; reverse, CGTACATGGGAGCGTC). Primers were provided by Eurofins (Ebersberg, Germany). 

RT-PCR results were related to medical history, specific treatments for COVID-19, signs of conjunctival inflammation (follicular reaction and hyperemia), and corneal fluorescein staining. The time-lapse from the first diagnosis of SARS-CoV-2 infection (first positive swab) and the method of administration of supplemental oxygen was also evaluated.

### 2.3. Cell and Virus

Vero cells (ATCC CCL-81, Manassas, VA, USA) were cultured in Eagle’s Minimal Essential Medium (EMEM) in addition with 10% Fetal Bovine Serum (FBS), 2 mM L-glutamine, and 100 IU/mL of penicillin–streptomycin solution at 37 °C/5% CO_2_. SARS-CoV-2 (clinical isolate, kindly provided by Lazzaro Spallanzani Hospital, Rome, Italy) was grown on Vero cells as already reported (doi: 10.3390/microorganisms9081550, doi: 10.3390/v13071263) in a biosafety level 3 (BSL3) containment laboratory. All the materials used for cell culture were acquired by Microtech Srl, Naples, Italy.

### 2.4. Plaque Assay

Each of the nine samples was inoculated on Vero cells and the potential cytopathic effect (CPE) was evaluated after 48 h. Then the supernatant was collected and titrated on the cell monolayer overlaid with 3% carboxymethylcellulose. After 2 days post-infection, cells were fixed with 4% formaldehyde, stained with 0.5% crystal-violet, and viral plaques were counted. The percentage of infection was calculated with respect to the positive control (CTRL+) consisting of Vero cells infected with SARS-CoV-2.

### 2.5. Statistical Analysis

Statistical analysis was carried out using GraphPad Prism version 8 software (GraphPad Software Inc., San Diego, CA, USA) by analysis of variance (ANOVA) and Dunnett’s test. Differences were considered statistically significant with *p* < 0.05.

## 3. Results

### 3.1. Patients Clinical Data

Eighteen eyes from nine patients (7 males/2 females) were examined; mean age: 73.4 ± 10.4 years (from 63 to 93). The clinical data of the patients examined are shown in Table 1.

### 3.2. RT-PCR

The ocular surface findings and the conjunctival swab SARS-CoV-2 RT-PCR results are reported in Table 2.

**Figure 1:**
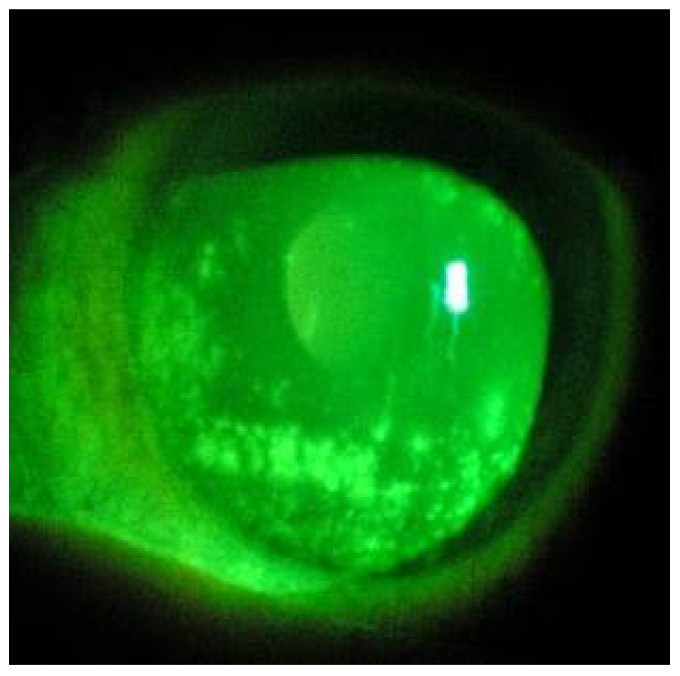
Corneal appearance with cobalt blue light filter pointing out epithelial corneal defects after instillation of sodium fluoresceinate 2%.

The presence of SARS-CoV-2 RNA in the conjunctival swabs were found on 7 of 9 patients; in 6 of these 7 patients with SARS-CoV-2 ocular involvement, the swabs were positive in both eyes; only one patient was found to have the virus in one eye. Therefore a total of 13/18 eyes (72%) tested positive for SARS-CoV-2 was registered. The prevalence of bilateral conjunctivitis complicating the course of COVID-19 was 78%. Corneal epithelial defects (punctate keratopathy) (Figure 1) were detected in 9 eyes of 7 subjects, all of which positive to SARS-CoV-2 molecular detection from ocular swabs. Three patients with SARS-CoV-2 positive eye swab had diabetes mellitus; two had oncological diseases. The seven subjects with SARS-CoV-2 virus ocular involvement had undergone treatment with a total face mask or oxygen helmet in the last week (average 7 ± 3.7 days); the two patients with negative conjunctival swabs were treated only with a high-flow nasal cannula (average 6.5 ± 0.5 days).

### 3.3. Plaque Assay 

**Figure 2:**
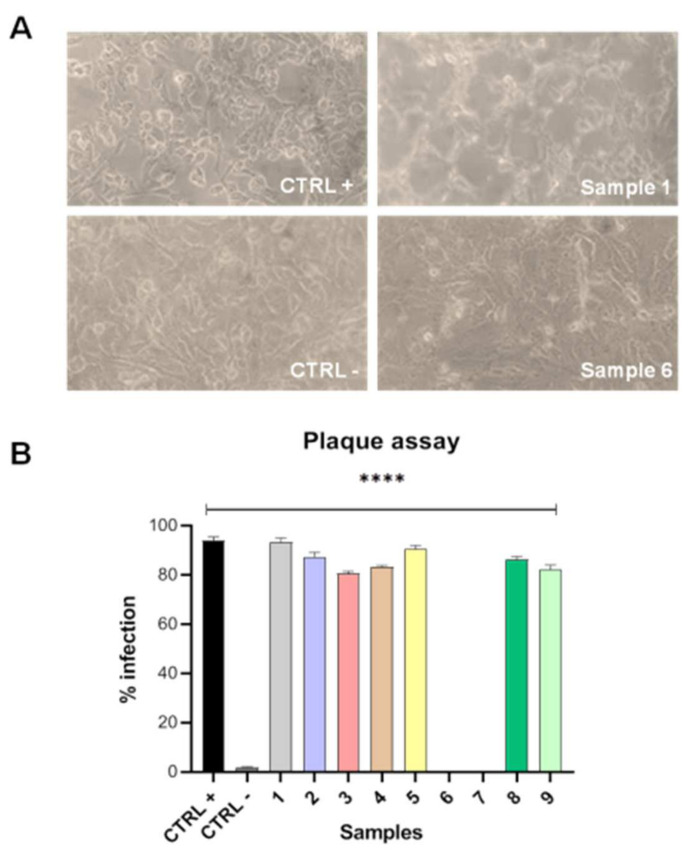
Plaque assay. (**A**) CPE observed after samples inoculation on Vero cell monolayer. Sample 6 did not show any CPE as CTRL—(not infected cells), meanwhile sample 1 exhibited rounded and detached cells, typical features observed after SARS-CoV-2 infection in vitro (CTRL+); (**B**) viral load quantification after 48 h post-infection. CTRL+ refers to cells infected with SARS-CoV-2. Differences were calculated by ANOVA and Dunnett’s test. **** *p* < 0.0001.

After evaluating the presence of SARS-CoV-2 RNA in the conjunctival swabs through RT-PCR, their viral load was determined by plaque assay. Each sample was inoculated on a Vero cell monolayer and CPE was observed after 48 h (Figure 2A). 

SARS-CoV-2-positive samples by RT-PCR (patients 1–5, 8 and 9) showed a strong CPE, meanwhile, on the contrary, negative samples (patients 6 and 7) did not exhibit any CPE. The viral load of each sample was evaluated by titrating the surnatant collected 48 h post-infection on Vero cell monolayer compared to CTRL+ (cells infected with SARS-CoV-2) (Figure 2B). The highest viral load referred to patients whose swab was positive in both eyes and with the major grade of conjunctival hyperemia (patients 1 and 5). On the other side, SARS-CoV-2-negative samples by RT-PCR were also confirmed through plaque assay since they did not show any viral plaques (patients 6 and 7).

## 4. Discussion

SARS-CoV-2 has been detected in the tears and in conjunctival specimens collected from infected patients [9], suggesting that the ocular surface and the tear liquid might represent a potential route for SARS-CoV-2 infection [10,11]. Considering that the nasolacrimal duct links the lacrimal sac of the eye to the nasal cavity, the COVID-19 could enter from the respiratory tract and infect the eye later [12]. The mechanism of SARS-CoV-2 cell entry depends on the viral S proteins binding to cellular receptors and on S protein priming by host cell proteases [6,13]. A multicenter study, which documented potential risk factors for SARS-CoV-2 transmission in patients requiring intubation, reported that unprotected eye contact with secretions from infected patients was the most predictive variable for transmission to healthcare workers [14]. A similar case was reported in the year 2020, when a doctor working in Wuhan, who wore an N95 mask, but did not wear eye protection, was subsequently infected with SARS-CoV-2 and presented marked conjunctival hyperemia [3]. These observations highlighted the importance of using protective eyewear as an integral part of personal protective equipment to avoid infection [14,15].

In the present study, a high percentage of positive tests for SARS-CoV-2 in tears was found (72%), higher than that reported in the literature (10–15% of cases) [15]. The reason for this result could be related to the differences in patients’ conditions, such as the use of facial respiratory devices; generally, not severe patients were included in the past studies.

Viral infection of the ocular surface affected over ¾ of the COVID-19 patients examined, and in all cases, at least one eye had corneal epithelial defects. Patients with SARS-CoV-2 ocular involvement had COVID-19 diagnosed 3 to 25 days earlier (mean 13.7 ± 6.4 days), while in the two cases with negative eye swabs, the diagnosis of the viral disease was made 11 and 9 days before, respectively. In all cases of SARS-CoV-2 ocular swab positivity, treatment with a total face mask or helmet had been applied. Five of these patients suffered from diabetes mellitus or systemic oncologic pathologies. The two patients with negative conjunctival swabs had diabetes mellitus.

These preliminary seem to suggest that corneo-conjunctival involvement could not be correlated with the presence of immunodeficiency or diabetes mellitus, nor with the severity of COVID pneumonia, nor with the time of onset of the systemic disease.

Some studies show the occurrence of mask-associated ocular dryness and irritation and exposure keratopathy by incomplete or inadequate eyelid closure; these effects are very frequent in all mask wearers, so patients and providers must be aware of these potential risks [16,17]. Frequent conjunctival and corneal involvement exposes the patients admitted to intensive care, with low immunological defenses, to viral contamination and bacterial superinfections. Patients with COVID-19 pneumonia have a high concentration of the virus in the breath, which can easily contaminate an ocular surface already suffering from dry eye or with corneal defects [17,18].

We assume that the high frequency of ocular involvement could be linked to direct contamination by the use of respiratory facial devices; the eyes of the two patients who had not used face masks were not positive for the virus. In patients 2–4, 8 and 9, hyperemia was detected in both eyes with a higher degree in the eye with epithelial defects. These differences are probably related to a different degree of viral exposure of the ocular surface and to different basic conditions, such as dry eye phenomena or pre-existing or concomitant corneal traumatic micro-lesions [12]. The same reasons can explain the presence of one positive eye and the other negative in patient n. 8; otherwise, this result could be due also to a false-negative test for low viral load.

The original aim of the research was to detect the virus in the conjunctiva and tear fluid, and the presence of signs of keratoconjunctivitis in patients hospitalized for SARS-CoV-2 pneumonia. The possible correlation with the use of facial masks should be confirmed on a larger number of patients by carrying out the conjunctival swab before and after the use of these devices in order to evaluate their effect on the viral colonization of the ocular surface.

However, the data obtained suggest the opportunity to implement ocular protection procedures during the use of facial respiratory devices, in order to avoid the ocular spread of the virus.

## 5. Conclusions

The high percentage of ocular involvement found in patients undergoing treatment with oxygen total face mask or helmet seems to suggest possible viral contamination of the ocular surface from the respiratory tract in patients with COVID-19 pneumonia. 

The present study suggests that exposure of unprotected eyes to aerosols containing high concentrations of SARS-CoV-2 virus could cause keratoconjunctivitis, much more frequently than reported in the literature so far.

The main limitation of the study is the small number of patients. Further studies are therefore necessary to verify this causal association in patients suffering from acute respiratory SARS-CoV-2 infection, the role of concomitant immunological pathologies, and of ocular surface disorders.

## Figures and Tables

**Table 1:** Clinical data of the examined patients.

Patient	Gender	Age	Clinical History	Days Elapsed from the First Swab Positive for SARS-CoV-2	Systemic Therapy	Oxygen Supplement Mode
1	Male	64	T2DM, Hypertension	25 days	Piperacillin/Tazobactam Enoxaparin, Dexamethasone, Omeprazole, Amlodipine, Insulin	Helmet CPAP
2	Female	93	Hypertension, LBBB	14 days	Clarithromycin, Dexamethasone, Omeprazole, ARBs	Venturi mask
3	Female	64	Ovarian neoplasia Colostomy	9 days	Clarithromycin, Betametasone, Pantoprazole, Albumin, Furosemide	Face mask CPAP
4	Male	65	T2DM, Hypertension	3 days	Clarithromycin, Enoxaparin, Dexamethasone, Pantoprazole, Amlodipine, Metformin	Venturi mask
5	Male	63	Heart failure	12 days	Enoxaparin, Azytromicin, Amlodipine, Dexamethasone, Pantoprazole	Face maskCPAP
6	Male	79	Hypertension, T1DM, CVD	11 days	Bisoprolol, Amlodipine, Insulin, Enoxaparin, Pantoprazole	Nasal Cannula
7	Male	68	Hypertension, T2DM, Psoriasic arthritis	9 days	Azytromicin, Enoxaparin, Insulin, Dexamethasone, Pantoprazole, ACE-inhibitor/tiazide	Nasal Cannula
8	Male	82	Hypertension, COPD, Prostatic neoplasia	17 days	Azytromicin, Enoxaparin, Amlodipine, Dexamethasone, Pantoprazole, Silodosin	Venturi mask
9	Male	83	Hypertension, T2DM, Kidney neoplasia	16 days	Piperacillin/Tazobactam Enoxaparin, Dexamethasone, Pantoprazole, Nebivolol, Rapid insulin, Albumin, Amlodipine	Helmet CPAP

**Table 2:** Conjunctival hyperemia according to Efron grading scale for contact lenses complications (grade 0: normal; grade 1: trace; grade 2: mild; grade 3: moderate; grade 4: severe) and conjunctival swab results with SARS-CoV-2 RT-PCR test of each eye examined with the relative threshold cycle. N/A: not applicable.

Patient		Conjunctival Hyperemia (Efron Scale)	Corneal Fluorescein Test Result	RT-PCR-SARS-CoV-2 Conjunctival Swab	Ct
1	Right eye: Left eye:	Grade 4 Grade 3	Positive positive	positive positive	18.83 18
2	Right eye: Left eye:	Grade 2 Grade 3	Negative positive	positive positive	22.76 21.9
3	Right eye: Left eye:	Grade 2 Grade 3	Negative positive	positive positive	27.66 27.05
4	Right eye: Left eye:	Grade 4 Grade 3	Positive negative	positive positive	25.16 24.07
5	Right eye: Left eye:	Grade 3 Grade 3	Positive positive	positive positive	20.18 19.12
6	Right eye: Left eye:	Grade 0 Grade 1	Negative negative	negative negative	N/A N/A
7	Right eye: Left eye:	Grade 2 Grade 0	Negative negative	negative negative	N/A N/A
8	Right eye: Left eye:	Grade 3 Grade 3	Positive negative	positive negative	24.88 25.12
9	Right eye: Left eye:	Grade 3 Grade 4	Negative positive	positive positive	28.02 27.66
